# 3D car-detection based on a Mobile Deep Sensor Fusion Model and real-scene applications

**DOI:** 10.1371/journal.pone.0236947

**Published:** 2020-09-03

**Authors:** Qiang Zhang, Xiaojian Hu, Ziyi Su, Zhihong Song

**Affiliations:** 1 Jiangsu Key Laboratory of Urban ITS, Southeast University, Nanjing, Jiangsu Province, People's Republic of China; 2 Jiangsu Province Collaborative Innovation Center of Modern Urban Traffic Technologies, Southeast University, Nanjing, Jiangsu Province, People's Republic of China; 3 School of Transportation, Southeast University, Nanjing, Jiangsu Province, People's Republic of China; 4 Nanjing Traffic Safety Research Institute, Nanjing, Jiangsu Province, People's Republic of China; 5 Anhui Keli Information Industry Co., Ltd, Hefei, Anhui Province, People's Republic of China; Tongii University, CHINA

## Abstract

Unmanned vehicles need to make a comprehensive perception of the surrounding environmental information during driving. Perception of automotive information is of significance. In the field of automotive perception, the sterevision of car-detection plays a vital role and sterevision can calculate the length, width, and height of a car, making the car more specific. However, under the existing technology, it is impossible to obtain accurate detection in a complex environment by relying on a single sensor. Therefore, it is particularly important to study the complex sensing technology based on multi-sensor fusion. Recently, with the development of deep learning in the field of vision, a mobile sensor-fusion method based on deep learning is proposed and applied in this paper——Mobile Deep Sensor Fusion Model (MDSFM). The content of this article is as follows. It does a data processing that projects 3D data to 2D data, which can form a dataset suitable for the model, thereby training data more efficiently. In the modules of LiDAR, it uses a revised squeezeNet structure to lighten the model and reduce parameters. In the modules of cameras, it uses the improved design of detecting module in R-CNN with a Mobile Spatial Attention Module (MSAM). In the fused part, it uses a dual-view deep fusing structure. And then it selects images from the KITTI’s datasets for validation to test this model. Compared with other recognized methods, it shows that our model has a fairly good performance. Finally, it implements a ROS program on the experimental car and our model is in good condition. The result shows that it can improve performance of detecting easy cars significantly through MDSFM. It increases the quality of the detected data and improves the generalized ability of car-detection model. It improves contextual relevance and preserves background information. It remains stable in driverless environments. It is applied in the realistic scenario and proves that the model has a good practical value.

## Introduction

As we know, visual intervention has a significant influence on drivers' behaviour [1–2]. After the implementation of driverless technology, this phenomenon may be alleviated. In the field of driverless technology, the use of Light Detection and Ranging (LiDAR) point cloud to make up for the deficiencies of visual technology and improve the accuracy of environmental detection will be the key developing directions for environmental awareness of unmanned vehicles in the future [3]. Moreover, 3D data, which is produced by various 3D sensors such as LIDAR and stereo cameras, has been widely deployed by industry leaders such as Google, Uber, Tesla, and Mobileye, for mobile robotic applications such as autonomous driving and humanoid robots [4]. Notably, the traffic accidents of unmanned vehicles often occurin an instant, which makes it necessary to obtain traffic data with high resolution [5]. Point cloud data, which is composed of reliable depth information, can provide accurate location and shape characteristics for scene understanding, such as object recognition and semantic segmentation [4]. To gather high-precision information about the suroundings of unmanned ground vehicles, LiDAR is frequently used to collect large-scale point clouds [6].The evolution and improvement of LiDAR and cameras have increased the quality and quantity of the gathered data [7], which can dispose of the conundrum well.

In the unmanned environment-aware device, LiDAR and cameras have their own advantages and disadvantages [8]. The advantage of the camera is that the cost is low, and there are many people who use the camera to develop algorithms, and the technology is relatively mature. The disadvantage of the camera is that it is relatively limited by ambient light. The advantage of the LiDAR is that it has a long detecting range and can accurately acquire the three-dimensional information of the object. In addition, its stability is quite high and the robustness is good. However, the current cost of LiDAR is high, and the final shape of the product has not yet been determined. Practices have proved that compared with single-sensor system, multi-sensor technology can enhance the survivability of the system, improve the reliability and robustness of the whole system, increase the real-time performance of the system and information utilization, and enhance the credibility of the data in solving problems such as detecting, tracking and recognizing [9].

**Fig 1:**
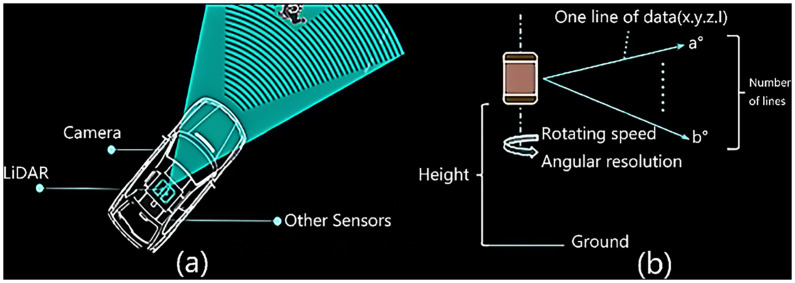
Principle of the on-board LiDAR and camera imaging. (a) shows the location of the LiDAR and camera on a car. (b) shows specific scanning range of LiDAR.

The non-scanning imaging system is a new type of imaging radar that appeared in the 1990s. It can overcome the problems of low frame rate, small field of view and large volume due to the lack of mechanical scanning devices [10]. It has high frame rate, wide field of view and volume. The principle of LiDAR point cloud imaging system is based on the laser beam scanning the target scene, receiving the laser radiation reflected by the scene, generating a continuous analog signal, and reducing the image into a real-time target scene [11]. Principle of LiDAR point cloud imaging is as Fig 1. LiDAR point cloud is a kind of active detecting technology, which can acquire the three-dimensional spatial information of the target accurately and quickly. Due to its unique technical advantages in object recognition, classification, high-precision 3D imaging and measurement, the application scope and development prospect of LiDAR are quite broad. Recently,3D imaging LiDAR is gradually developed from single point scanning to small array scanning, line array sweep and array flash imaging. At the same time, the single photon detection technology is becoming mature and the detection sensitivity is getting higher and higher. With the development of modern detection technology, it is more and more inclined to the fusion detection of various sensors and the development of 3D imaging is also inclined to the combination of active and passive imaging to obtain more abundant target’s information [12]. LiDAR point cloud have broad applied prospects in the field of traffic engineering. In terms of transportation, Zhang Z. Y. [5] proposed that a LiDAR sensor was installed at the roadside for data collection, which is convenient for collecting data. Wu [13] proposed a new mode of retrieving data using LiDAR that roadside LiDAR deployment provides a solution to obtain the real-time high-resolution micro traffic data of unconnected road users for the connected-vehicle road network. Zhang Mingfang [14] proposed a new method for classifying an oncoming vehicle's turning behavior at intersection using 3D LIDAR. Javanmardi [15] proposed that state-of-the-art localization approaches adopt 3D Lidar to observe the surrounding environment and match the observation with a priori known 3D point cloud map for estimating the position of the vehicle within the map. Dimitrievski [16] present a novel 2D-3D pedestrian tracker designed for applications in autonomous vehicles using LiDAR datasets. Lim [17] proposed a LiDAR information based authentication mechanism for V2V communication, to authenticate vehicles locally without involvement of a trusted authority and infrastructures. Zeng [18] present a real-time three-dimensional (RT3D) vehicle detection method that utilizes pure LiDAR point cloud to predict the location, orientation, and size of vehicles.

In fact, it is very easy for the camera to find the same object, such as tracking people or tracking the car, but for LiDAR, it is very difficult to identify whether the front and rear frames are the same car and the same pedestrian. LiDAR has one advantage: if it can be told by the camera that the two frames are the same object, then LiDAR can be used to know the object in the interval between the two frames about the speed of motion and the displacement of the motion. This is very critical, because only the tracking of moving objects can make some predictions. During the driving process, it has to know the moving state of the surrounding objects at all times. For unmanned driving, in addition to estimating the position of the vehicle and navigating, it is also necessary to track and predict surrounding objects and moving objects, which is very meaningful.

As mentioned above, relying on only one kind of sensor is difficult to accurately detect targets, if there are other factors that interfere with its normal operation. Nowadays, many researchers have conducted a comprehensive study on sensor fusion, and their purpose is to make up for the shortcomings of single sensor through sensor fusion. Ali MAH [19] proposed that the sensor fusion algorithm of LiDAR and cameras is used to remove noises and uncertainties from sensors' data and provide optimum measurements for path planning. Dimitrievski [16] employed Camera and LiDAR data fusion to solve the association problem where the optimal solution is found by matching 2D and 3D detections to tracks using a joint log-likelihood observation model. Asvadi [20] proposed a multi-mode car-detection system that integrates data from 3D-LIDAR and color cameras with data from LIDAR and cameras in three modes. Then it was combined to improve car-detection. Many investigators have studied the methods of sensor fusion to improve the detecting efficiency and accuracy. Chen Xiaozhi [21] proposed Multi-View 3D networks (MV3D), a sensory-fusion framework that takes both LIDAR point cloud and RGB images as inputs and predicts oriented 3D bounding boxes. Mousavian [22] present a method using deep learning for 3D object detection and pose estimation from a single image, which first regresses relatively stable 3D object properties using a deep convolutional neural network and then combines these estimates with geometric constraints provided by a 2D object bounding box to produce a complete 3D bounding box. Danfei Xu [23] proposed a novel fusion network using deep learning, which predicts multiple3D box hypotheses and their confidences, using the input 3D points as spatial anchors. Stanislas [24] described a novel probabilistic sensor fusion framework aimed at improving obstacle detection accuracy and classification of various targets experienced in the Maritime RobotX Challenge. Frossard [25] proposed a novel approach to tracking by detection that can exploit both cameras as well as LIDAR data to produce very accurate 3D trajectories, which is an end-to-end learning method of multi-sensor 3D tracking by detection.

It usually uses previous car-detecting methods to identify cars. The problems with the methods are as follows. (a)The detection of the first step usually depends on the characteristics and rules of artificial design, such as setting some thresholds, surface normals, etc., and the generalized ability is poor [26]. (b)Multi-stage processing means that complex errors may occur—clustering and classification are not based on a certain context, and environmental information around the target is missing [27]. (c)This type of method is unstable for the computational time and accuracy of single-frame LiDAR scanning, which is contrary to the safety requirements (stable, small variance) in autonomous driving scenarios [28].Therefore, in recent years, many ideas of detecting targets based on deep learning have been proposed. Mobile Deep Sensor Fusion Model (MDSFM) is proposed in this paper, which uses convolutional neural network to extract features of images and implement fusion of the LiDAR and camera in car-detection. It can improve accuracy and efficiency through settling the deficiencies in the traditional car-detection using MDSFM.

## Materials and methods

### Three-dimensional data processing

#### Datasets

**Fig 2:**
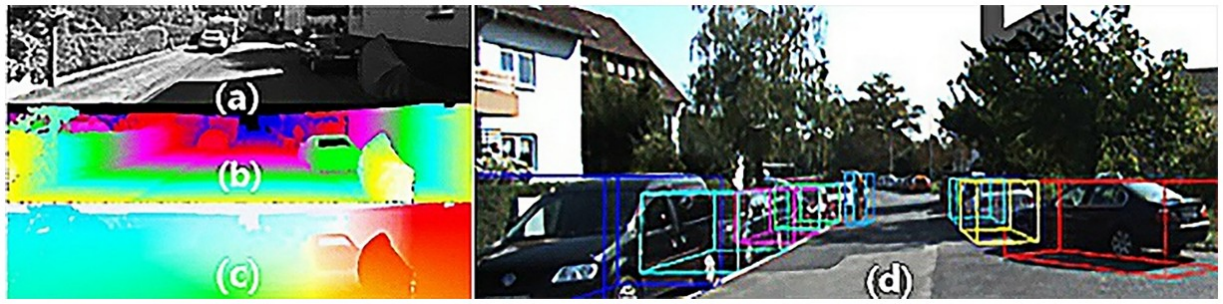
The depths and stereo images of the datasets. (a) shows an RGB image. (b) and (c) show different depths of LiDAR scanning images. (d) shows a 3D labeled image in the perspective of the camera.

**Fig 3:**
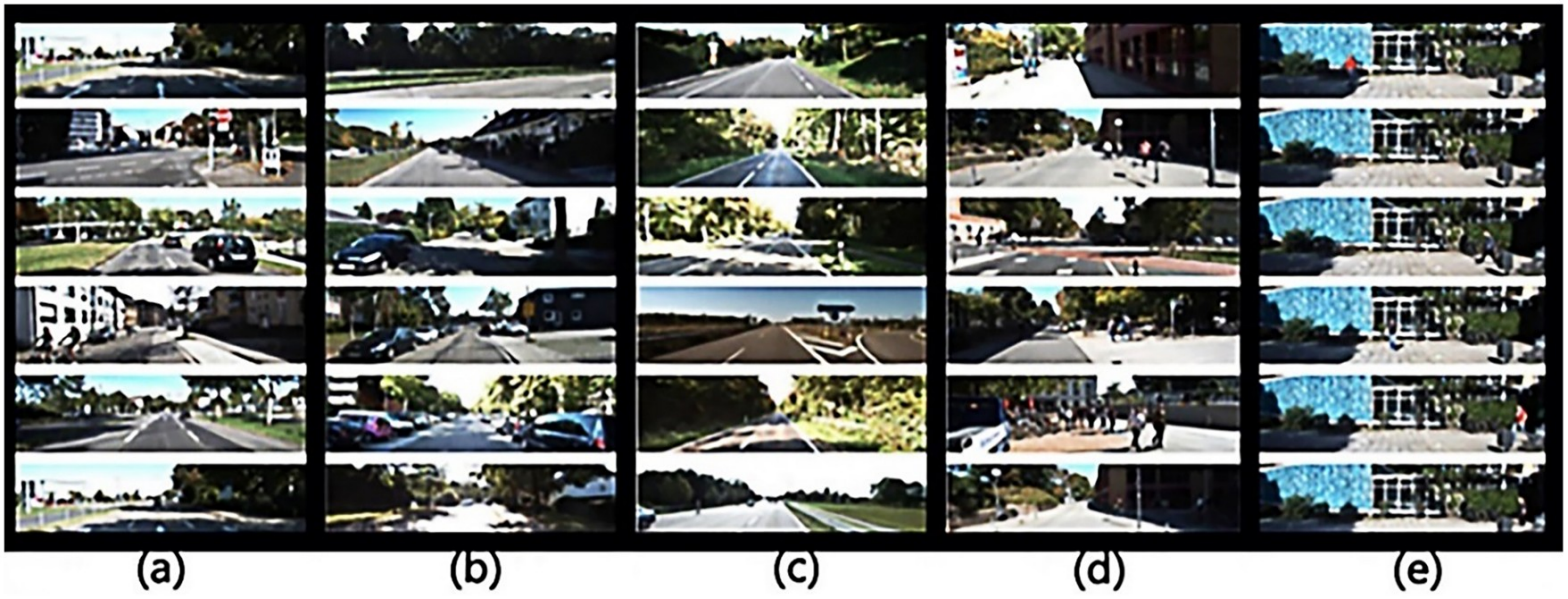
The scenes in the datasets. (a) shows the ‘Road’ scenes. (b) shows the ‘City’ scenes. (c) shows the ‘Residential’ scenes. (d) shows the ‘Campus’ scenes. (e) shows the ‘Person’ scenes.

The datasets are released by KITTI (A project of Kartsruhe Institute of Technology and Toyota Technological Institute at Chicago) and the typical samples of the datasets are shown in Fig 2. The datasets contain over 93 thousand depth maps with corresponding raw LiDAR scans and RGB images, aligned with the "raw data" of the KITTI datasets, including the 3D and BEV datasets for validation [29]. The datasets are used to evaluate the performance of computer vision such as stereo, optical flow, visual odometry, 3D object detection, and 3D tracking in the driving environment and we supplement information suitable for programs and practices. As shown in Fig 3, the entire KITTI datasets contain images collected from cities, campuses, etc. Each image has up to 15 cars evenly, as well as various degrees of occlusion and truncation. The entire datasets consist of 389 pairs of stereo images and optical flow maps, a 39.2 km visual ranging sequence, and images of more than 200k 3D labeled objects, which are sampled and synchronized at a frequency of 10 Hz.

#### The pretreatment of LiDAR point cloud data

In order to improve the speed of the algorithm, many algorithms do not directly utilize the 3D point cloud data, but project the point cloud data to a 2D plane and then process it. Common two-dimensional data forms are Range Image and Elevation Image. The main reasons are that the accuracy of object detection directly in three dimensions is not high enough, and currently path planning and vehicle control generally only consider the motion of the car body in a two-dimensional plane. From the testing results, the algorithms are better than object detection in three-dimensional space. The object detection method directly acting in three-dimensional space has also made a breakthrough in recent years. It extracts the features of the three-dimensional point cloud with the invariance of orders through some operators, and then processes directly in the three-dimensional point cloud through a specially designed network. The advantage of this type of method is that it can perform indiscriminate detection of objects in any positions and directions of the entire three-dimensional space. The idea is novel but limited by the capabilities of the algorithm itself, the capabilities of the hardware device, and the actual application scenarios.

**Fig 4:**
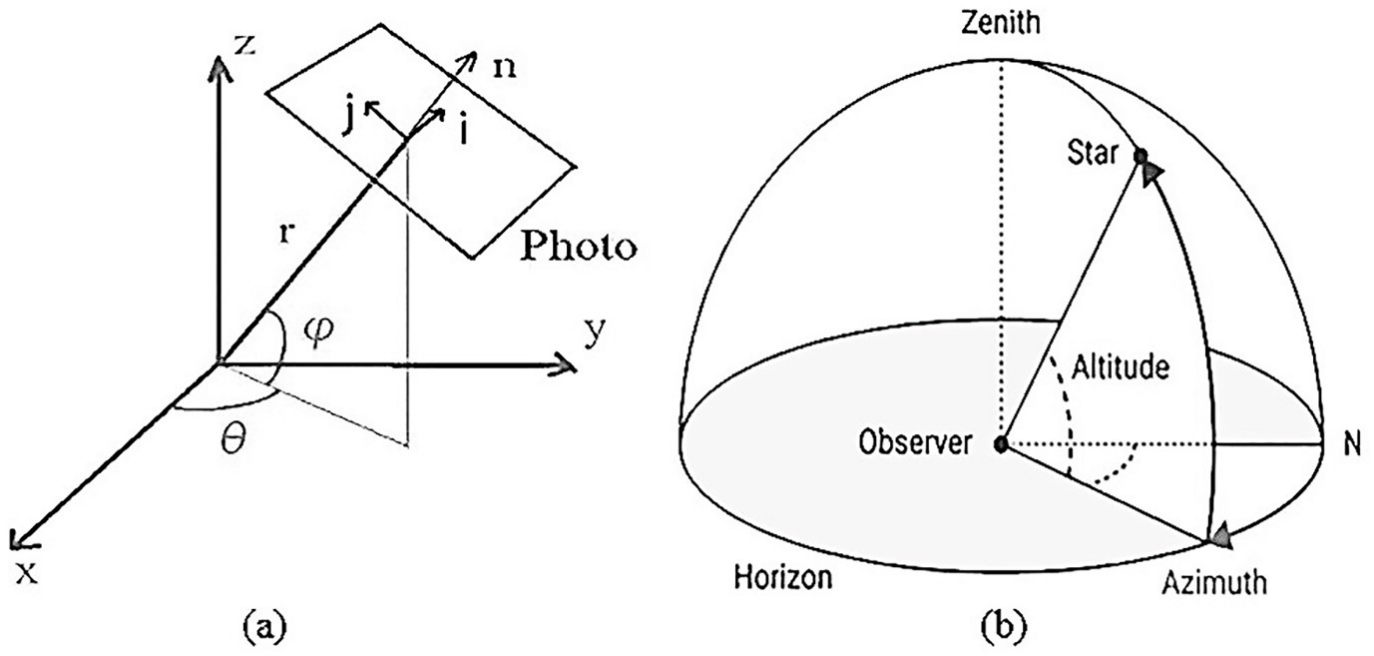
The spherical projection. (a) shows coordinates from 3D target to 2D target. (b) shows observer’s perspective.

The traditional designs of CNN are mostly used for two-dimensional image-pattern-recognition, and the formats of the three-dimensional point cloud data do not conform to this mode, and the point cloud data is sparse and irregular, and this extraction of feature is unfavorable [30]. Therefore, before inputting data to CNN, the data is first spherically projected to a two-dimensional and dense data. The spherical projection is as Fig 4.

Generally speaking, the azimuth is the angle with respect to the true north direction, but in the LiDAR coordinate system, the azimuth is the angle with respect to the *x* direction (directly in front of the vehicle). The calculation of φ and θ can be expressed as:

$$\left\{ \begin{array}{l} \theta =\text{arcsin}\frac{z}{\sqrt{x^{2}+y^{2}+z^{2}}}\\ \phi =\text{arcsin}\frac{y}{\sqrt{x^{2}+y^{2}}} \end{array}\right.$$ (1)

Where (x, y, z) are the coordinates of each point in the three-dimensional point cloud. So for every point in the point cloud, you can calculate (θ, φ) by its (*x*, *y*, *z*). It projects the points in the three-dimensional coordinate system to a spherical coordinate system. This spherical coordinate system is already a two-dimensional coordinate system, but for the sake of understanding, it differentiates its angle to obtain a two-dimensional cartesian coordinate system. The coordinate system can be expressed as:

$$\{\begin{array}{l} i=\frac{\theta }{\delta \theta }\\ j=\frac{\phi }{\delta \phi } \end{array}$$ (2)

**Fig 5:**
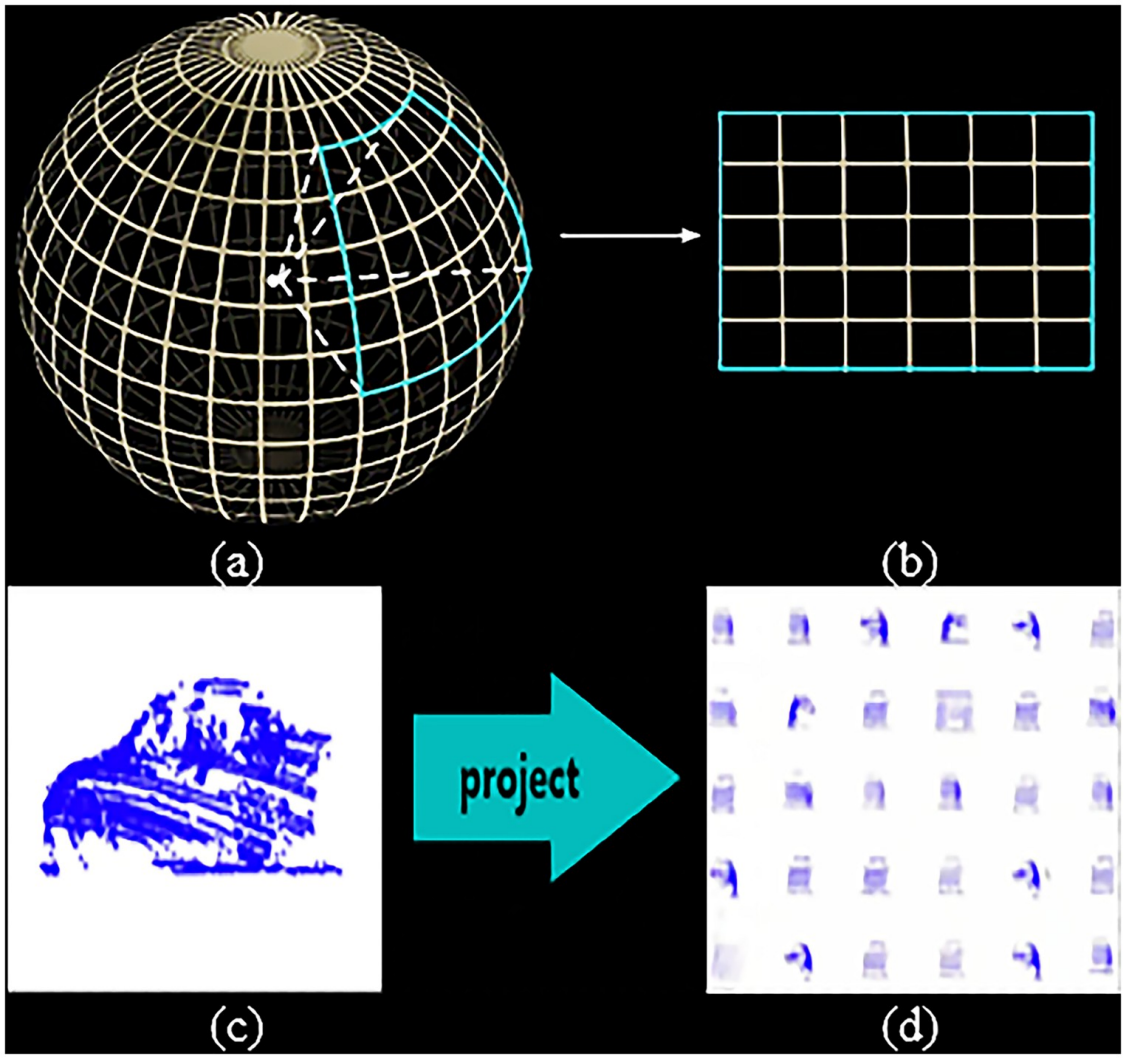
The projection of three-dimensional graphics to two-dimensional graphics. (a) shows Cartesian Coordinate System. (b) shows that a size of (H, W, C; where C = 5) tensor is obtained. (c) shows a three-dimensional object. (d) shows a 2D trainable dataset.

Then, each point in the spherical coordinate system can be represented by a point in a Cartesian Coordinate System, as Fig 5. Through such a layer transformation, it projects the position (*x*, *y*, *z*) of any point in the three-dimensional space to the position (*i*, *j*) of a point in the two-dimensional coordinate system. It extracts each point in the point cloud. Five features (*x*, *y*, *z*, intensity, range) are placed in the corresponding two-dimensional coordinates (*i*, *j*).

### Sensor fusion based on MDSFM

Compared to previous sensor fusion models, this model is more lightweight, which uses only two inputs to complete the fusion of LiDAR and camera. Compared with previous sensor fusion models that have three inputs, the dual-view MDSFM directly reduces the amount of calculation under the premise of ensuring accuracy. It is also one of our jobs to connect LiDAR and camera modules. The images processed by LiDAR and camera modules are used as inputs for 3D Box Regressor.

**Fig 6:**
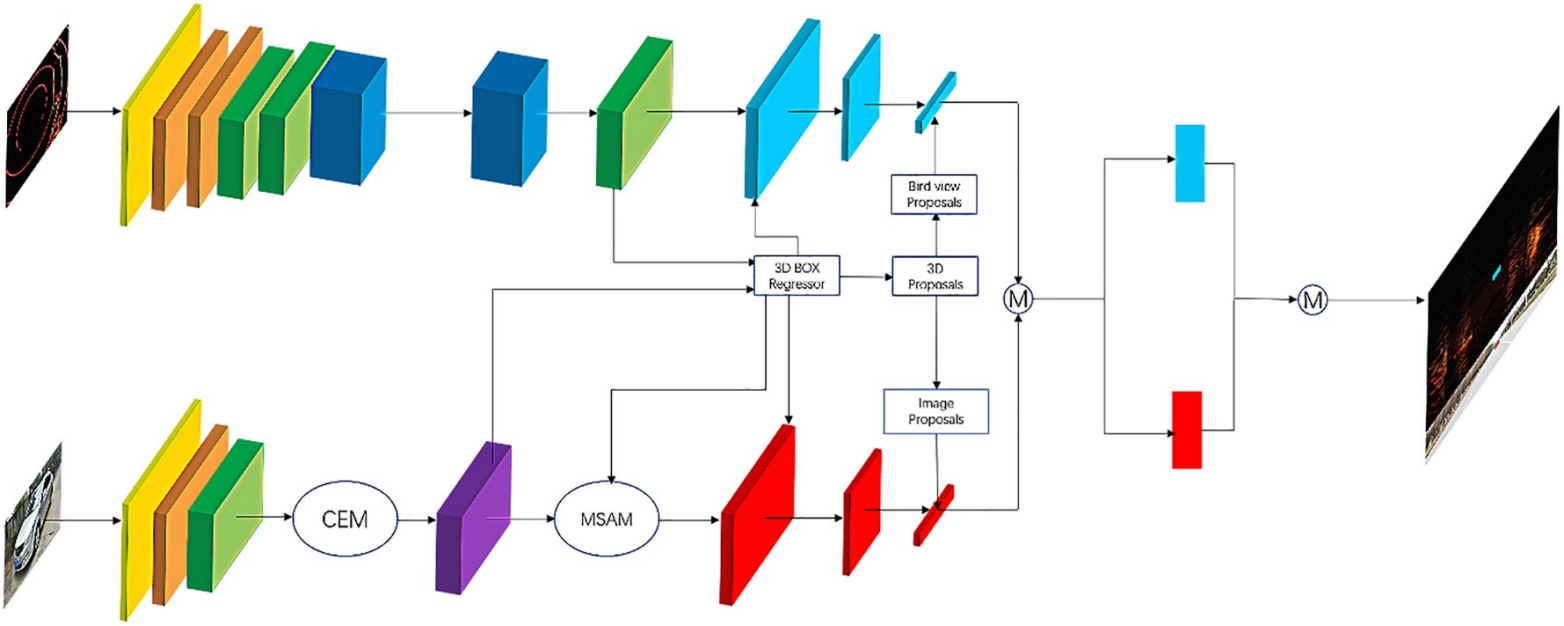
The structural network of Mobile Deep Sensor Fusion Model.

**Fig 7:**
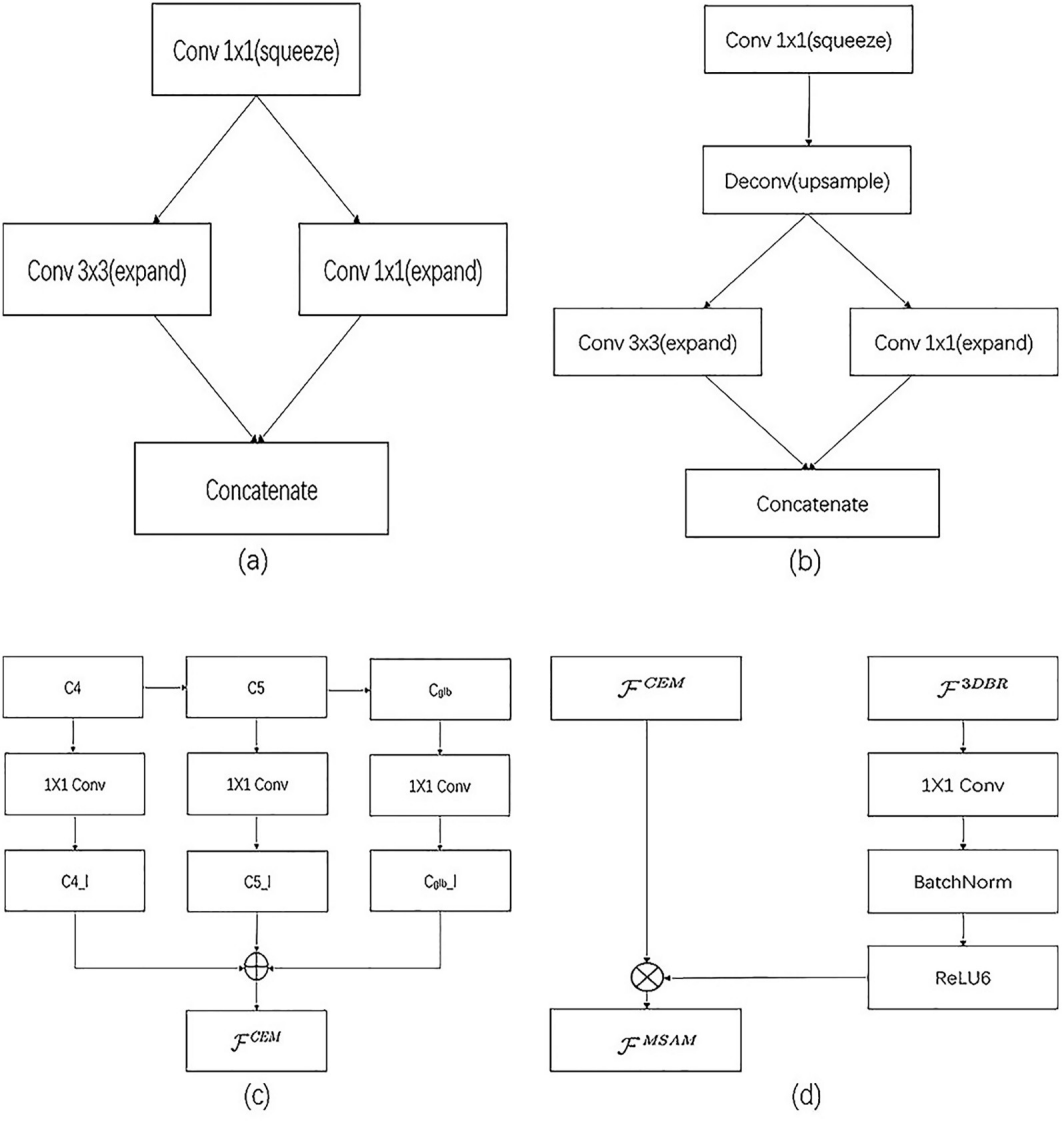
The key modules of sensor fusion method. (a) shows the structure of fireModules. (b) shows the structure of fireDeconvs. (c) shows a Context Enhancement Module. (d) shows a Mobile Spatial Attention Module.

As shown in Fig 6, in the view of LiDAR, the input point cloud is processed by CNN, which includes fireModules, and then it outputs results according to 3D box regressor and 3D proposals, and then processed by the deep fusion module of MDSFM. It applies a convolutional network on the 2D point map and predicts 3D boxes densely from the convolutional feature maps. It is more efficient, and on the basis of ensuring the efficiency, we improve the accuracy of detection. The significance of using fireModules is that it helps to reduce the parameters of the convolutional network but capable of achieving the accuracy of AlexNet model [31], which is lightweight and efficient. Under the same conditions, compared to AlexNet, the parameters of squeezing model are 50x less than AlexNet, but the performance is similar to AlexNet [31]. As shown in Fig 7, the fireModule mainly consists of two layers of convolution operations: one is the squeezed layer with 1x1 convolutional kernels, the other is the expanded layer with 1x1 and 3x3 convolutional kernels. The fireDeconv has a more Deconv layer than the fireModule. Of course, the structure can be compressed to make the model smaller. In order to reduce the number of parameters and computation, it replaces convolution and deconvolution with fireModules and fireDeconvs in this module.

In the view of the camera, the input images are the front view. This module uses the revised detecting design of the lightweight R-CNN. Moreover, it consists of a Context Enhancement Module (CEM) and a Mobile Spatial Attention Module (MSAM). The key idea of CEM that leverages semantic and context information from multiple scales is to aggregate multi-scale local and global information to produce more discriminating features and the receptive field size plays an important role in CNN models [32]. CNNs can only capture information inside the receptive field. Thus, a large receptive field can leverage more context information and encode long-range relationship between pixels more effectively. This is crucial for the localization subtask, especially for the localization of large objects. In CEM, feature maps of three scales are merged: C4, C5, and Cglb [33]. And Cglb is the global feature information stemmed from global average pooling on C5. By using local and global information, CEM effectively expands the receptive field and refines it. Moreover, compared to the previous FPN structure, CEM involves only two 1x1 convolutions and c-layers, which is speedier in terms of computations. The key idea of MSAM is to use the information from 3D box regression (3DBR) to refine the charactermatic distribution of the feature map. And MSAM uses the information trained in 3D box regression to refine the feature distribution. 3D box regression is trained to recognize foreground regions under the supervision of ground truths. Therefore, the intermediate features in 3D box regression can be used to distinguish foreground features from background features. MSAM accepts two inputs: the intermediate feature map from 3DBR $F^{3DBR}$ and the thin feature map from CEM $F^{CEM}$.

Due to the lightweight nature of MSAM, it is more suitable for mobile devices. The output of MSAM $F^{MSAM}$ is defined as:

$$F^{MSAM}=F^{CEM}\frac{ReLU6(F^{3DBR}+3)}{6}$$ (3)

Where *ReLU6* is a piecewise linear function that limits the maximum output to 6. The *ReLU6* can be implemented in many deep learning frameworks, and at the same time, the loss of the precision value is reduced during quantization. Each 3D box is parameterized by (*x*, *y*, *z*, *l*, *w*, *h*), which are the center and size of the 3D box. 3DRB is regressed to **t** = (Δ*x*, Δ*y*, Δ*z*, Δ*l*, Δ*w*, Δ*h*), which are the offsets of the center and size in the 3D box.

However, it is necessary to take into account the accuracy of this real-time model. In extracting features, it uses the optimized module named Position Sensitive ROI Align (PSRol Align) [34]. To improve the accuracy of the model, it uses PSRol align to optimize the results. The main improvement is to reduce errors caused by two-step quantizations. The operation is to keep the boundary coordinate values of the ROI and the boundary values of all units in each ROI in the form of floating points. In each unit, the pixel values of a fixed number of sampling points at a fixed position are calculated for average pooling. The main idea is to manually introduce the information of the position in the charactermatic aggregation, thereby effectively improving the sensitivity of deeper neural networks to the information of the car’s position. And computations are performed directly on the entire picture, which also greatly optimizes the operating speed of the network.

As shown in Fig 6, to enable more interactions among features of the intermediate layers from the view of LiDAR and camera, it uses the dual-view deep fusion, which can be expressed as:

$$\{\begin{array}{l} f_{0}=f_{LiDAR}⊕f_{CAMERA}\\ f_{l}=H_{l}^{LiDAR}(f_{l-1})⊕H_{l}^{CAMERA}(f_{l-1})\\ \forall l=1,\cdots ,L \end{array}$$ (4)

Where all of *f* are features and {**H**_*l*_, *l* = 1, ⋯, *L*} are feature transformation functions and ⊕ is a join operation (e.g., concatenation, summation).

### Implement a program with the Robot Operating System on the experimental car

Robot Operating System (ROS) is to provide a flexible framework for developers, which contains a list of tools, libraries, and conventions. At the same time, ROS can also provide middleware similar to operating systems for heterogeneous computing clusters.

**Fig 8:**
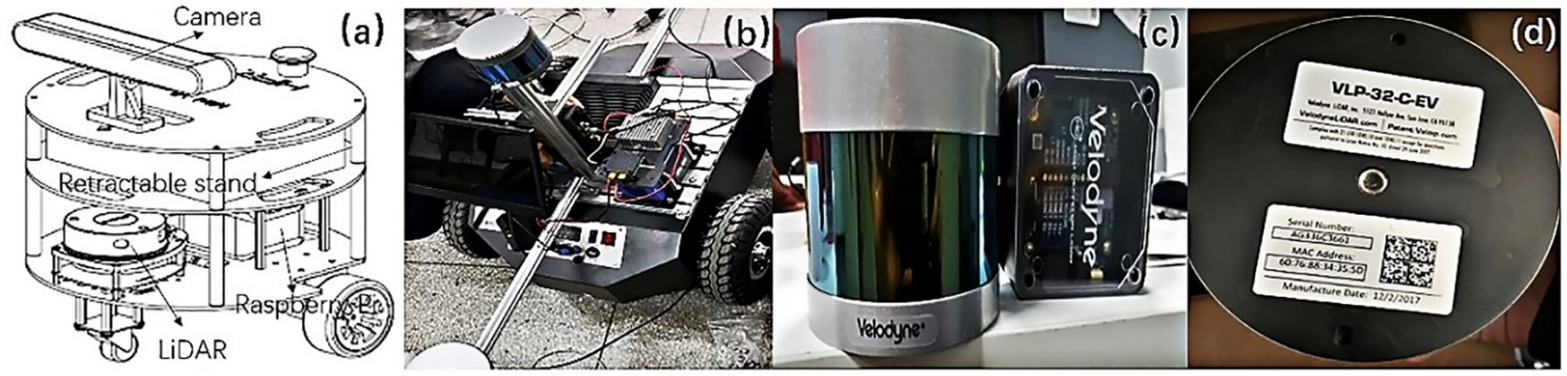
The construction of the robot. (a) shows the main construction of a robot. (b) shows the installed part of the robot. (c) and (d) show Velodyne LiDAR used in the experiment.

**Fig 9:**
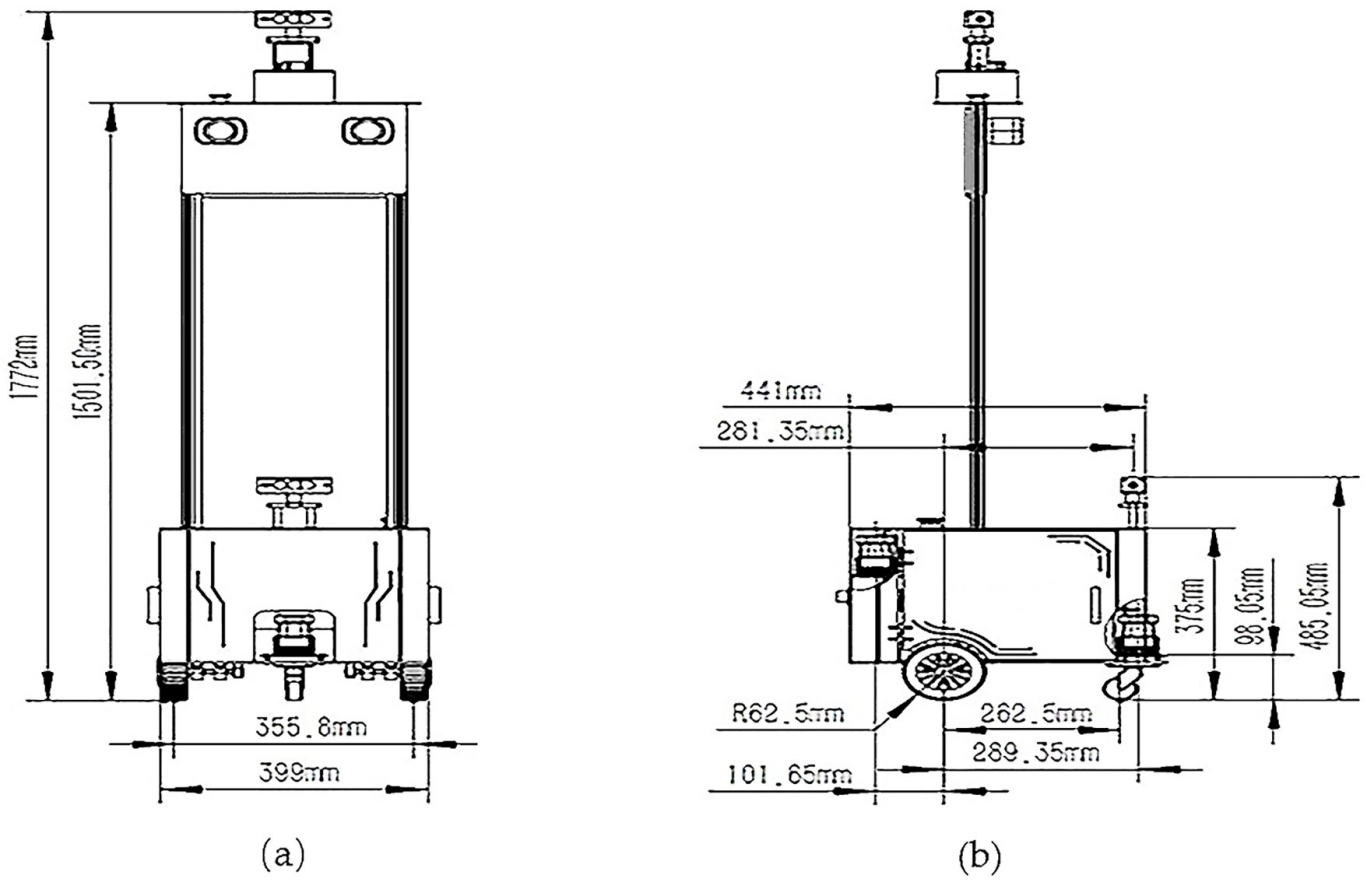
The parameters of the robot. (a) shows the front view of the robot. (b) shows the lateral view of the robot.

**Fig 10:**
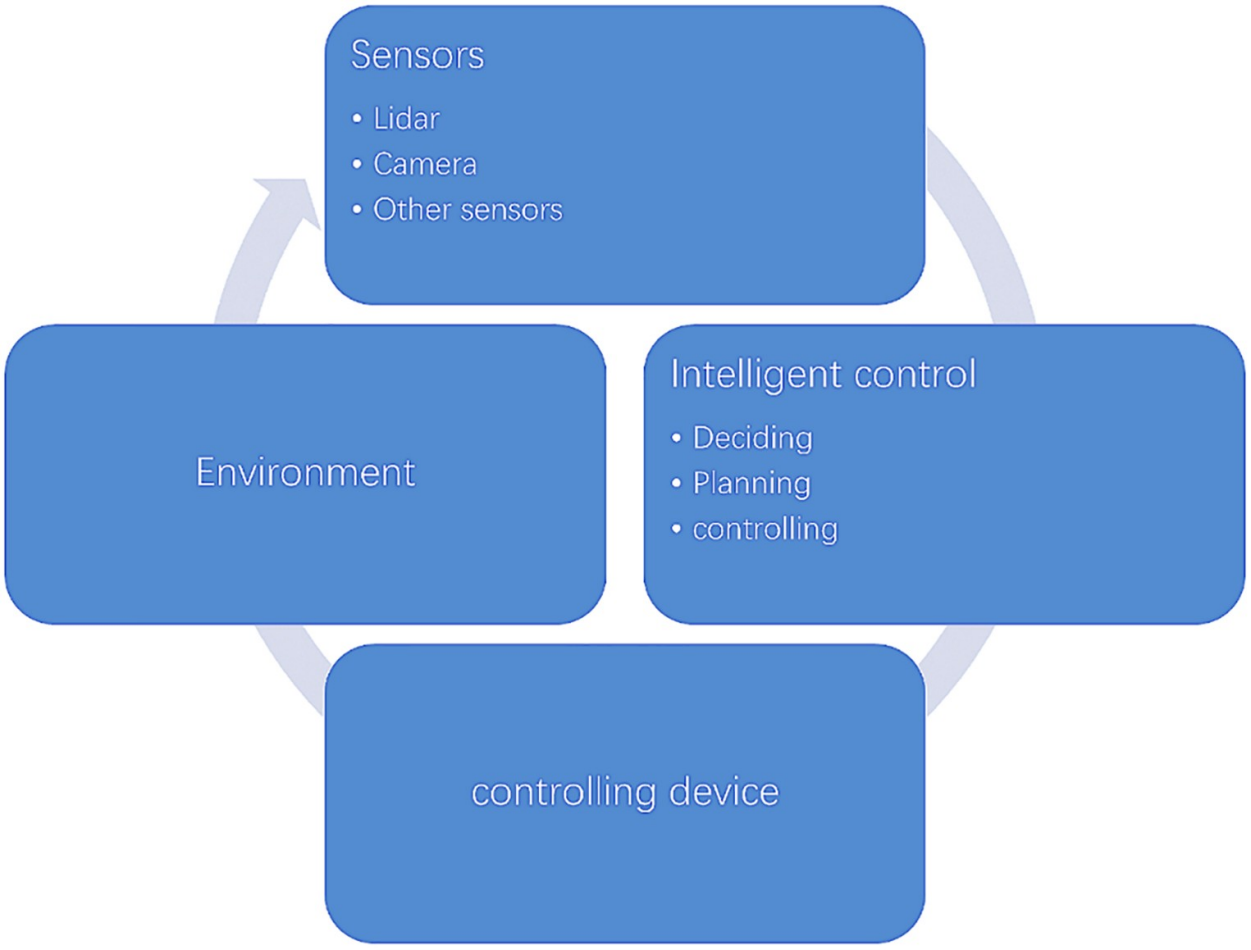
The operating process of ROS.

As Fig 8(a) shows, the top is the camera and Lidar is under the camera. As Fig 9 shows, the camera is installed above the LiDAR and using retractable stands can change its height. The camera is responsible for navigation, avoiding obstacles and collecting images. The Raspberry Pi is the core of the robot and controls the LiDAR and camera. The computer with GPU for high-performance computation is connected to the Raspberry Pi via network, and the model can be tested normally after debugging according to the data transmitted by Raspberry Pi. As Fig 8(b) shows, the straight rod connected to the LiDAR can be freely telescoped to meet the height of LiDAR. It needs to be adjusted for the height and angle of the camera and LiDAR to ensure that cars can be detected, and also calibrate the camera and LiDAR to ensure accurate detection of the vehicle. As Fig 8(c) and 8(d) show, it uses the Velodyne VLP-32C LiDAR, which can track 32 targets simultaneously. Furthermore, the VLP-32C LiDAR has 32 channels, an effective range of 200 meters, and a double echo mode that produces approximately 1.2 million 3D point cloud coordinates per second in a view of 360° horizontal field and a 40° vertical field. The dense channel distribution of the ULTRA Puck near the horizontal angle gives it a higher resolution over longer distances. It operates in a dual returning mode and can capture larger details of 3D images. The small size and light weight make this sensor ideal for automotive applications. The detected images and point clouds are sent to the computer connected to the Raspberry Pi and at the same time it calls the model for recognition. The operating process of the ROS project is shown in Fig 10.

## Results and discussion

### Result on KITTI’s benchmark

It usually evaluates performance using the mature KITTI’s benchmark of 3D object detection. Far objects are thus filtered based on their bounding box height in the image plane. As only objects also appearing on the image plane are labeled, objects in no car areas do not count as false positives. It notes that the evaluation does not take care of ignoring detections that are not visible on the image plane—these detections might rise to false positives. For cars it requires an 3D bounding box overlap of 70%. Evaluated Criteria and difficulties are defined as follows according to the convention of KITTI [29].

1. Easy (E): Min. bounding box height: 40 Px, Max. occlusion level: Fully visible, Max. truncation: 15%.
2. Moderate (M): Min. bounding box height: 25 Px, Max. occlusion level: Partly occluded, Max. truncation: 30%.
3. Hard (H): Min. bounding box height: 25 Px, Max. occlusion level: Difficult to see, Max. truncation: 50%.

**Fig 11:**
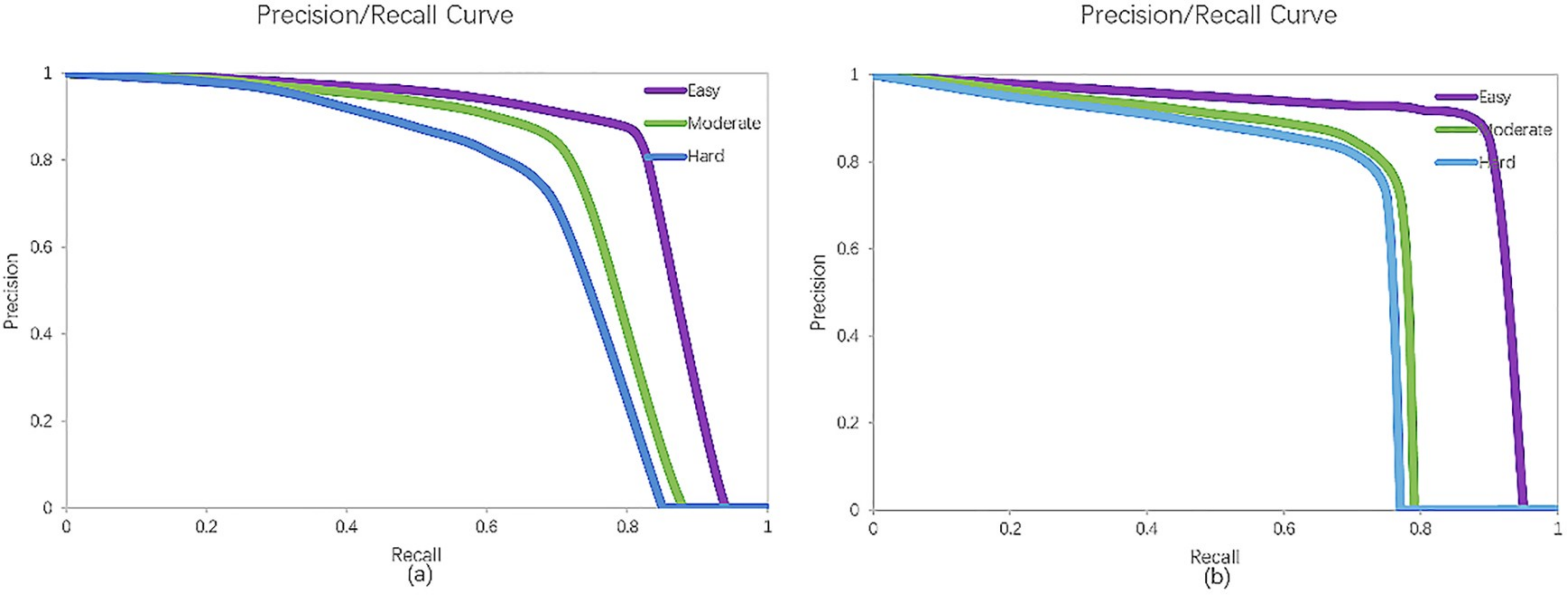
The evaluated results of the Mobile Deep Sensor Fusion Model. (a) shows the evaluated result of 3D car-detection. (b) shows the evaluated result in the bird’s eye view.

The performance of car-detection is evaluated. It derives the precision-recall curves as shown in Fig 11. The precision (P), the recall (R) and the Intersection over Union (IoU) are used as the indexes of the performance, and are respectively defined as:

$$\{\begin{array}{l} \text{precision}=\frac{TP}{TP+FP}\\ \text{recall}=\frac{TP}{TP+FN}\\ IOU=\frac{TP}{TP+FP+FN} \end{array}$$ (5)

Where True Positive (TP) is the number of predicting positive samples as positive samples; True Negative (TN) is the number of predicting negative samples as negative samples; False Positive (FP) is the number of predicting negative samples as positive samples; False Negative (FN) is the number of predicting positive samples as negative samples.

The main configuration of hardware used for training is: CPU (Core i7-9700) and GPU (Titan X). The processor is a GPU (TITAN X) according to the KITTI’s benchmark.

**Fig 12:**
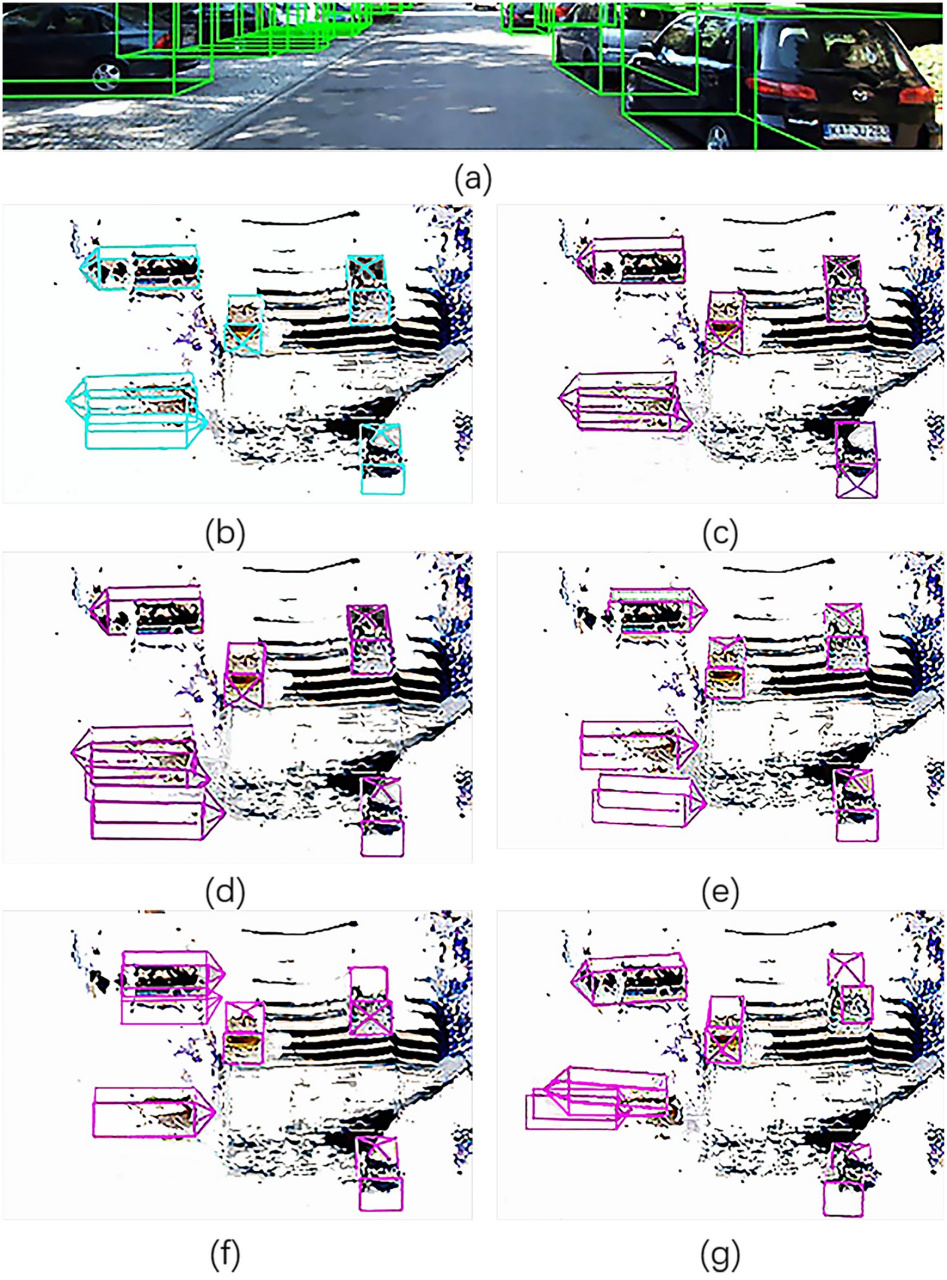
Comparison of the methods for car detection. (a) only shows the camera’s view. (b) shows Ground Truth. (c)-(f) show performances of the other methods. (g) shows the performance of our method.

It selects images from the KITTI’s datasets for validation to test this model. It evaluates the mentioned models on the KITTI’s benchmark of detecting objects that provides 7,481 images for training and 7,518 images for testing. The code is implemented by TensorFlow and Python. So before running the code, we need to install the TensorFlow-GPU. Based on the above warehouse, it calls a trained model to identify and fuse the input images of the LiDAR and camera, and concurrently it forms 3D bounding boxes in order to identify cars accurately as shown in Fig 12.

Average Precision (*AP*) is the area under the Precision-recall curve. 3D detecting results are evaluated by using the 3D and BEV *AP* values at 0.7 *IoU* threshold for the car-class. The other mentioned sensor-fusion methods are admittedly mature and have better performances of the recognized methods currently in the field of multi-view fusion and real-time 3D car-detection.

As shown in Table 1, it evaluates the performance of the method from the perspectives of Three-Dimensional (3D) car-detection and Bird's Eye View (BEV). In terms of 3D car-detection, *AP*_*3D*_ in column E of our method is 83.81%, which is higher than values of the other methods. *AP*_*3D*_ in column M of our method is 75.12%, which is less than the value of AVOD method, but higher than values of the other methods. *AP*_*3D*_ in column H of our method is 72.33%, which is less than the value of AVOD method but higher than values of the other methods. In the bird's-eye view, *AP*_*BEV*_ in column E of our method is 90.35%, which is higher than values of the other methods. *AP*_*BEV*_ in column M of our method is 78.20%, which is higher than the value of MV3D method. *AP*_*BEV*_ in column H of our method is 75.02%, which is higher than the value of MV3D method. The runtime of our method is 0.047 seconds in the same hardware and datasets, which is more efficient and has a reasonable real-time performance.

**Table 1:** Average Precision (AP) of car-detection (*IoU* = 0.7).

Method	*AP*_*3D*_ (%)			*AP*_*BEV*_ (%)			Runtime(s)
	E	M	H	E	M	H	
MV3D [21]	71.09	62.35	55.12	86.02	76.90	68.49	0.360
AVOD [35]	77.63	86.61	76.06	86.80	85.44	77.73	0.080
F-PointNet [36]	81.20	70.39	62.19	88.70	84.00	75.33	0.170
ContFuse [37]	82.54	66.22	64.04	88.81	85.83	77.33	0.060
MDSFM	83.81	75.12	72.33	90.35	78.20	75.02	0.047

### Result on ROS

**Fig 13:**
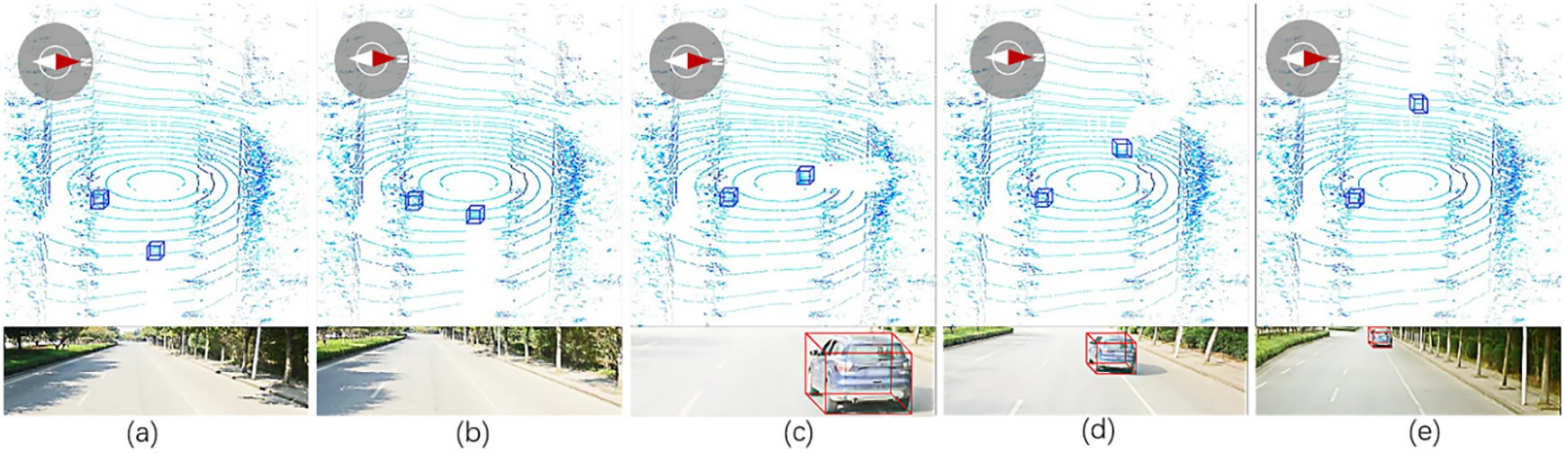
The recorded samples. (a) shows the image of Frame 1. (b) shows the image of Frame 50. (c) shows the image of Frame 100. (d) shows the image of Frame 150. (e) shows the image of Frame 200.

In this system, it uses the LiDAR and camera to collect information about the surrounding cars. Then it processes information and recognizes cars. The recorded samples are as Fig 13. The robot moves from east to west. During driving the robot, if the driving speed is too fast, bumps will occur and cause the phenomenon of unstable imaging. Therefore, it sets the driving speed of the experimental car at a slow speed. Moreover, if the height of the device is set too low, on the one hand, it is not conducive to detect other cars; on the other hand, it is dangerous that other moving cars may not see the experimental car and collide with it. Therefore, we set the height of the camera to 1.772 m and the angle is 12 degrees down the horizontal line. The details of each frame are summarized as Tables 2–4.

**Table 2:** Detailed information about general specifications.

Frame. name	general specifications			
	No.of frames	Scene condition	Robot’s situation	Obj. type
(a)	1	urban	moving	car
(b)	50	urban	moving	car
(c)	100	urban	moving	car
(d)	150	urban	moving	car
(e)	200	urban	moving	car

**Table 3:** Detailed information about camera's challenging factors.

Frame. name	Camera's challenging factors			
	Occlusion	Illumination variations	Obj.pose variations	Change in the obj. size
(a)	no	no	no	no
(b)	no	no	no	no
(c)	yes	no	yes	yes
(d)	yes	no	no	no
(e)	yes	no	no	no

**Table 4:** Detailed information about LiDAR's challenging factors.

Frame. name	LIDAR's challenging factors		
	No. of obj. points	Distance to the obj.	Velocity variations
(a)	low→high	far→near	no
(b)	high	near	no
(c)	high	near	no
(d)	high	near	no
(e)	high→low	near→far	yes

## Conclusions

The specific innovations and contributions are as follows:

Firstly, it does a data processing that projects 3D data to 2D data, which can form a dataset suitable for the model, thereby training data more efficiently.
Secondly, in the modules of LiDAR, it uses a revised structure to lighten the model and reduce parameters. In the modules of cameras, it uses the improved design of detecting module in R-CNN with a Mobile Spatial Attention Module (MSAM). In the fused part, it uses a dual-view deep fusing structure.
Thirdly, it selects images from the KITTI’s datasets for validation to test this model. Compared with other recognized methods, it shows that our model has a fairly good performance.

Finally, it implements a ROS program on the experimental car and our model is in good condition.

The results show that it can improve performance of detecting easy cars significantly through MDSFM, which takes advantages of both LiDAR and cameras. Moreover, it increases the quality of the detected data and improves the generalized ability of car-detection model. It improves contextual relevance and preserves background information. It remains stable in driverless environments. Ultimately, it is applied in the realistic scenario and proves that the model has a good practical value.

## Supporting information

S1 Fig Robot Operating System (ROS). (DOCX)

S2 Fig The RGB images. (DOCX)

S3 Fig The point clouds. (DOCX)
