# Supplementary figures and images for: 3D car-detection based on a Mobile Deep Sensor Fusion Model and real-scene applications
Source: PLoS One. 2020 Sep 3;15(9):e0236947. doi: 10.1371/journal.pone.0236947 (PMC7470372; doi:10.1371/journal.pone.0236947)

**
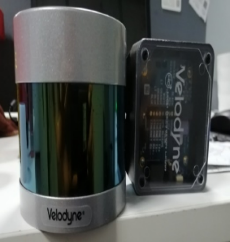

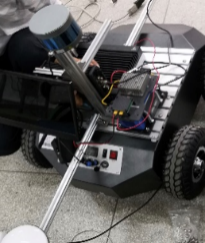

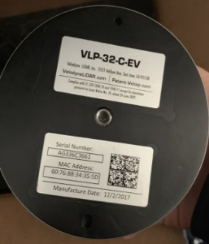

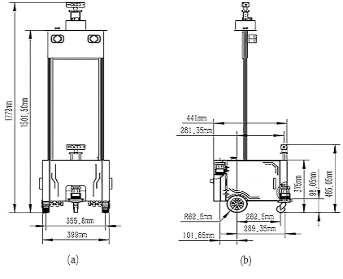

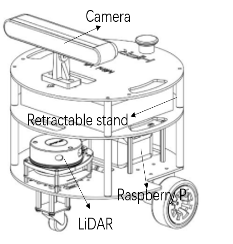
S1 Fig. Robot Operating System (ROS):**

Supplement: S1 Fig — (DOCX) [file pone.0236947.s001.docx]

**
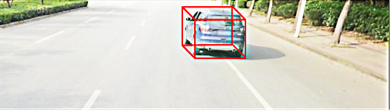

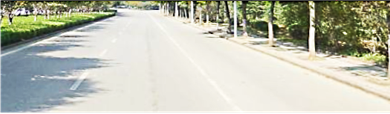

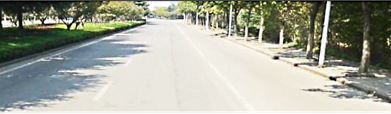

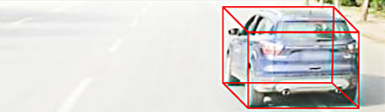
S2 Fig. The RGB images:**

**
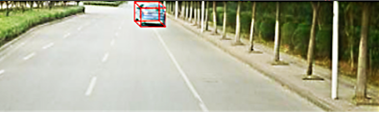
**

Supplement: S2 Fig — (DOCX) [file pone.0236947.s002.docx]

**
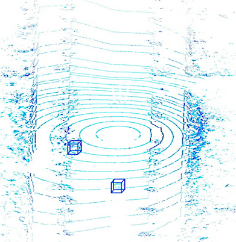

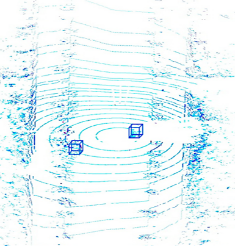

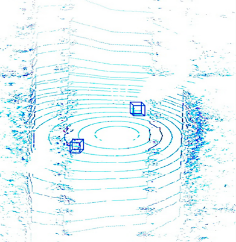

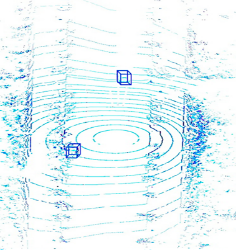

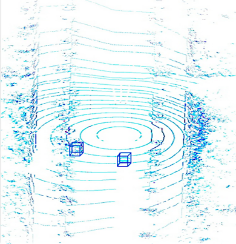
S3 Fig. The point clouds:**

Supplement: S3 Fig — (DOCX) [file pone.0236947.s003.docx]
